# Three New Indole Alkaloids from *Tabernaemontana divaricata*

**DOI:** 10.1007/s13659-018-0166-x

**Published:** 2018-05-12

**Authors:** Yan Deng, Mei-Fen Bao, Bao-Bao Shi, Jing Wu, Xiang-Hai Cai

**Affiliations:** 1 State Key Laboratory of Phytochemistry and Plant Resources in West China, Kunming Institute of Botany, Chinese Academy of Sciences, Kunming, 650201 People’s Republic of China; 2 University of Chinese Academy of Sciences, Beijing, 100049 People’s Republic of China; 3 Yunnan Key Laboratory of Natural Medicinal Chemistry, Kunming, 650201 People’s Republic of China

**Keywords:** *Tabernaemontana divaricata*, Monoterpene indole alkaloids, 3*α*-hydroxymethyl-ibogamine, 3*α*-acetatemethoxyl-ibogamine, 16*α*-hydroxyl-ibogamine, Apocynaceae

## Abstract

**Abstract:**

Three new monoterpene indole alkaloids, 3*α*-hydroxymethyl-ibogamine (**1**), 3*α*-acetatemethoxyl-ibogamine (**2**), 16*α*-hydroxyl-ibogamine (**3**) together with six known alkaloids were isolated from the branches and leaves of *Tabernaemontana divaricata* (Apocynaceae). The structures of these alkaloids were determined by spectroscopic analyses. All isolated compounds showed no significant cytotoxicity against SGC-7901 gastric cancer, HeLa, and A-549 lung cancer cell lines (IC_50_ > 20 µM).

**Graphical Abstract:**

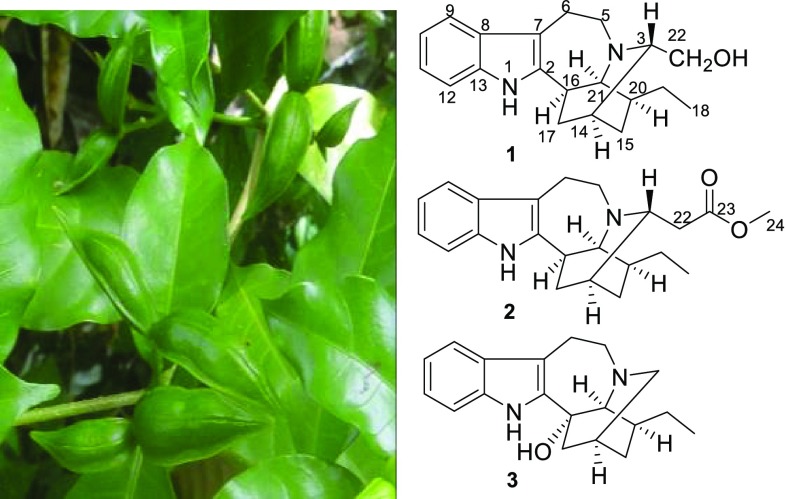

**Electronic supplementary material:**

The online version of this article (10.1007/s13659-018-0166-x) contains supplementary material, which is available to authorized users.

## Introduction

Alkaloids are unique natural products denoted by the presence of a nitrogen atom as part of a heterocyclic ring, constituting a highly diverse group of compounds. At present, more than 12,000 different alkaloids, all of them plant-derived, are known. To deal with this enormous diversity, these compounds are organized into dozens of structural related groups [1, 2]. Among this class of compounds, monoterpene indole alkaloids (MIAs) may account for a quarter of naturally occurring alkaloids. Both, their highly intricate chemical structures and pronounced pharmacological activities attracted many people from the research community to illuminate the structural diversity, bioactivities and biosynthetic pathways [3]. Species of the genus *Tabernaemontana* L. (Apocynaceae) are widely distributed in tropical and subtropical regions of Africa, Asia, North America, Pacific Islands, South America, including five species in China. This species are recognized as a rich source of MIAs [4], whereas the Iboga-type alkaloids ibogamine [5], voacangine [6], and coronaridine [7] are common in these species, hence, they can be regarded as chemical markers for the genus. These compounds feature a nitrogen-containing seven-membered ring which is linked to the indole system. As part of continuing search for bioactive alkaloids, three new alkaloids named as 3*α*-hydroxymethyl-ibogamine (**1**), 3*α*-acetatemethoxyl-ibogamine (**2**), 16*α*-hydroxyl-ibogamine (**3**), as well as six known alkaloids **4**–**9**, were isolated from the branches and leaves of *T. divaricata* (L.) R. Br. ex Roem. & Schult. The known alkaloids were identified as coronaridine (**4**) [8], isovoacangine (**5**) [9], taberdivarine G (**6**) [10], voacangine (**7**) [11], heyneanine (**8**) [12], coronaridine hydroxyindolenine (**9**) [13]. All of them belong to the iboga-type alkaloids. In the assessment of their bioactivities, the isolated alkaloids did not show significant activities against SGC-7901 gastric cancer, HeLa, and A-549 lung cancer cell lines (IC_50_ > 20 µM).

## Results and discussion

**Fig. 1:**
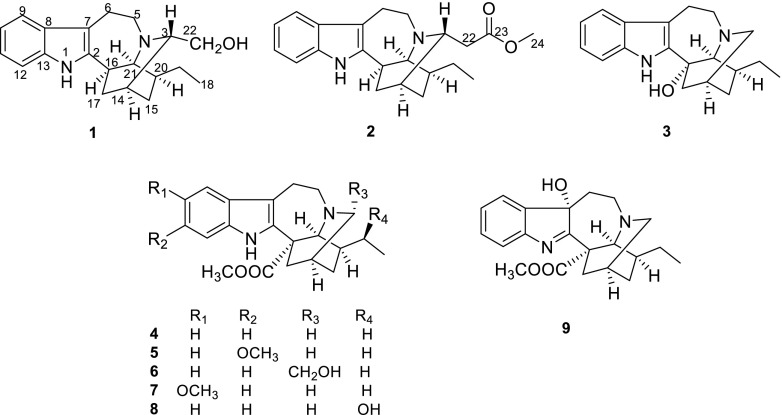
Structures of compounds **1**–**9**

The alkaloid fraction of *T. divaricata* was separated as described in experimental section to yield a total of nine compounds, including three new alkaloids **1**–**3** (Fig. 1). All compounds showed a positive response to Dragendorff’s reagent on TLC.

**Fig. 2:**
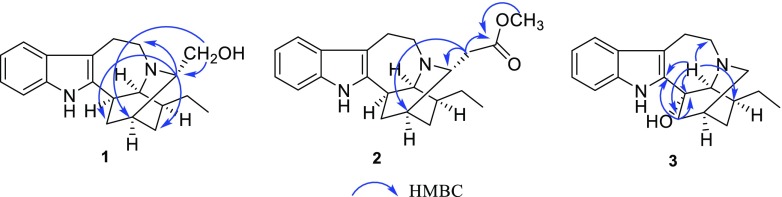
Key HMBC correlations of compounds **1**–**3**

**Fig. 3:**
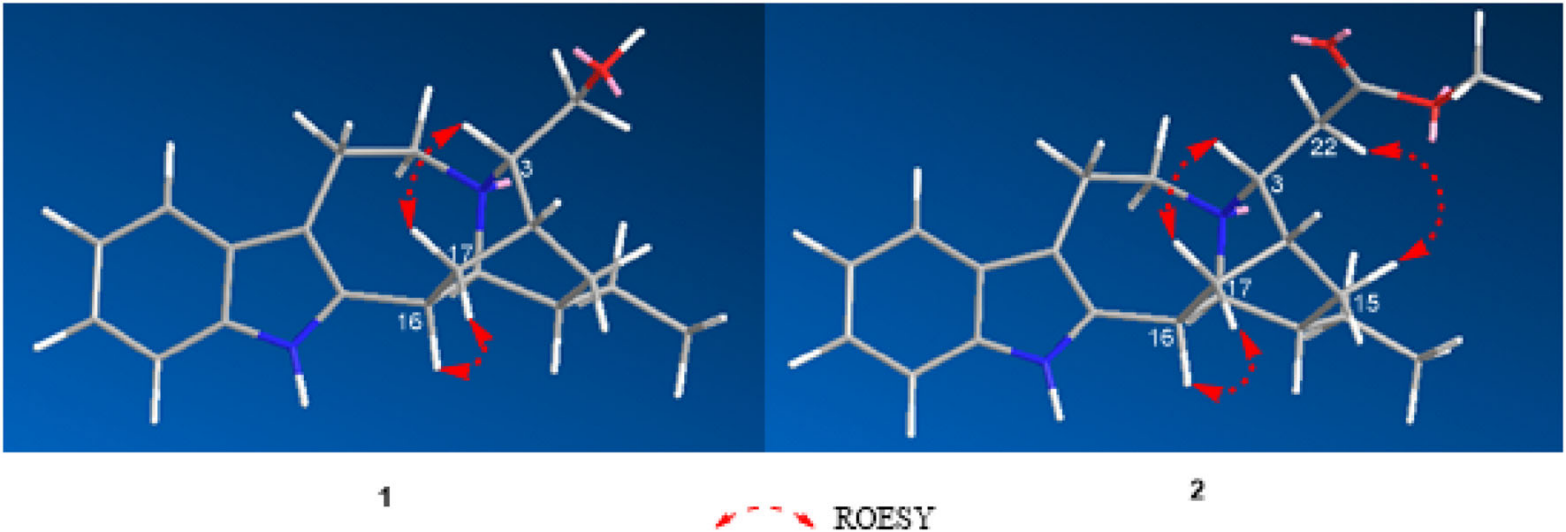
Key ROESY correlations of compounds **1** and **2**

Compound **1** was obtained as pale yellow amorphous powder. The UV absorption bands at 284, 227, and 214 nm suggest the presence of an indole chromophore [14]. The molecular formula of compound **1** was established as C_20_H_26_N_2_O with nine degrees of unsaturation by HRESIMS ([M + H]^+^ at *m/z* 311.2118). The ^1^H NMR data (Table 1) indicated an unsubstituted indole ring with signals at *δ*_H_ 7.38 (1H, d, *J* = 7.7 Hz), 6.94 (1H, t, *J* = 7.7 Hz), 6.99 (1H, t, *J* = 7.7 Hz), 7.22 (1H, d, *J* = 7.7 Hz), and one methyl signal at *δ*_H_ 0.87 (3H, t, *J* = 7.3 Hz). The ^13^C NMR and DEPT data (Table 1) revealed the presence of four quaternary carbons (*δ*_C_ 143.1, 136.2, 130.7, 108.6), nine methines (*δ*_C_ 121.1, 119.1, 118.3, 111.0, 59.8, 59.7, 42.3, 41.9, 27.9), six methylenes (*δ*_C_ 62.4, 53.8, 37.2, 27.9, 27.4, 21.3), and one methyl group (*δ*_C_ 12.2). The 1D NMR spectroscopic data of **1** were similar to those of ibogamine, except the presence of a hydroxymethyl group at C-3 and a methine carbon (*δ*_C_ 59.8) of C-3 in **1** instead of a methylene (*δ*_C_ 49.9) in ibogamine [5]. The HMBC correlations from *δ*_H_ 3.20 (H-3) to *δ*_C_ 27.4 (C-15), *δ*_C_ 37.2 (C-17), *δ*_C_ 53.8 (C-5), and from *δ*_H_ 3.48 (H-22) to *δ*_C_ 27.9 (C-14), *δ*_C_ 59.8 (C-3) confirmed this result (Fig. 2). Thus, the planar structure of compound **1** was determined. The relative configuration of **1** was determined on the basis of the ROESY spectrum, the correlation of H-17 (*δ*_H_ 2.17)/H-16, H-3/H-17 (*δ*_H_ 1.61), suggested H-3 was *β*-oriented (Fig. 3). Consequently, the structure of **1** was confirmed as shown in Fig. 1, and named 3*α*-hydroxymethyl-ibogamine (**1**).

**Table 1:** ^1^H (400 MHz) and ^13^C (125 MHz) NMR data for compounds **1**–**3** (*δ* in ppm, *J* in Hz)^a^

No.	**1**		**2**		**3**	
	*δ*_C_	*δ*_H_	*δ*_C_	*δ*_H_	*δ*_C_	*δ*_H_
1	–	9.70, s	–	9.70, s	–	9.74, s
2	143.1 s	–	142.9 s	–	145.6 s	–
3	59.8 d	3.20, dd (8.8, 4.1)	55.4 d	3.56, m	49.8 t	2.89, overlapped
5	53.8 t	3.00, m	53.0 t	2.99, m	54.4 t	3.12, ddd (12.5, 11.0, 4.2)
		3.53, ddd (14.4, 4.8, 2.2)		3.46, ddd (14.4, 4.8, 2.1)		3.27, m
6	21.3 t	2.63, ddd (16.4, 4.0, 2.2)	21.4 t	2.63, m	22.1 t	2.64, m
		3.37, m		3.35, ddd (17.1, 12.7, 4.8)		3.33, m
7	108.6 s	–	108.5 s	–	106.5 s	–
8	130.7 s	–	130.7 s	–	130.2 s	–
9	118.3 d	7.38, d (7.7)	118.4 d	7.39, d (7.7)	118.8 d	7.38, d (7.7)
10	119.1 d	6.94, t (7.7)	119.1 d	6.95, t (7.7)	119.0 d	6.95, t (7.7)
11	121.1 d	6.99, t (7.7)	121.2 d	7.00, t (7.7)	121.3 d	7.01, t (7.7)
12	111.0 d	7.22, d (7.7)	111.0 d	7.23, d (7.7)	111.5 d	7.36, d (7.7)
13	136.2 s	–	136.2 s	–	135.4 s	–
14	27.9 d	1.96, m	30.7 d	1.70, m	28.9 d	1.87, overlapped
15	27.4 t	1.40, overlapped	27.4 t	1.34, m	32.6 t	1.05, m
		1.59, overlapped		1.63, m		1.83, overlapped
16	41.9 d	3.11, ddd (11.7, 3.9, 2.0)	41.5 d	3.11, ddd (11.7, 3.7, 2.0)	74.7 s	–
17	37.2 t	1.61, overlapped	36.9 t	1.59, m	44.0 t	1.84, overlapped
		2.17, m		2.15, m		1.93, overlapped
18	12.2 q	0.87, t (7.3)	12.2 q	0.89, t (7.3)	12.1 q	0.87, t (7.4)
19	27.9 t	1.48, overlapped	28.1 t	1.45, m	28.1 t	1.49, m
20	42.3 d	1.50, m	42.2 d	1.52, m	35.1 d	2.27, m
21	59.7 d	2.85, overlapped	59.8 d	2.85, s	63.4 d	2.79, d (1.8)
22	62.4 t	3.48, dd (10.7, 8.8)	38.5 t	2.39, dd (14.8, 8.4)	–	–
		3.61, dd (10.7, 4.1)		2.60, d (5.0)		
23	–	–	173.2 s	–	–	–
24	–	–	51.5 q	3.60, s	–	–

^a^Data (*δ*) were measured in acetone-*d*_6_

Compound **2** was isolated as pale yellow amorphous powder. The ^1^H and ^13^C NMR data (Table 1) of **2** was very similar to those of **1**, except the presence of an additional –COOCH_3_ signal (*δ*_H_ 3.60, *δ*_C_ 51.5, *δ*_C_ 173.2) and the ^13^C NMR chemical shift at C-22 (*δ*_C_ 38.5) was different from that in compound **1** (*δ*_C_ 62.4). Moreover, compound **2** was assigned the molecular formula of C_22_H_28_N_2_O_2_, differing from **1** by addition of 42 daltons (da) and consistent with substitution of an OH by a COOCH_3_ group based on the analysis of HRESIMS ([M + H]^+^ at *m/z* 353.2223). This assumption was supported by HMBC correlations from *δ*_H_ 2.39 (H-22) to *δ*_C_ 30.7 (C-14), *δ*_C_ 55.4 (C-3), *δ*_C_ 173.2 (C-23), and from *δ*_H_ 3.60 (H-24) to *δ*_C_ 173.2 (C-23) (Fig. 2). Similar to **1**, the ROESY correlation of H-17 (*δ*_H_ 2.15)/H-16, H-3/H-17 (*δ*_H_ 1.59), H-15 (*δ*_H_ 1.34)/H-22 (*δ*_H_ 2.39) suggested that H-3 was *β*-oriented (Fig. 3). Hence, compound **2** was identified as 3*α*-acetatemethoxyl-ibogamine (**2**).

Compound **3** showed a molecular ion peak at *m/z* 297.1964 [M + H]^+^ (calcd. 297.1961) by HRESIMS, corresponding to the molecular formula C_19_H_24_N_2_O, which is 16 da more than that of the known alkaloid ibogamine [5]. Comparison of its 1D NMR data (Table 1) with those of ibogamine showed that the major differences were the signal due to C-17, C-21 were found to resonate at lower field (*δ*_C_ 44.0, 63.4) and a quaternary carbon (*δ*_C_ 74.7) in **3** substituted a methine in ibogamine. In addition, the HMBC correlations from the cross peaks of H-17 (*δ*_H_ 1.93)/C-16 (*δ*_C_ 74.7), C-2 (*δ*_C_ 145.6), and of H-21 (*δ*_H_ 2.79) /C-20 (*δ*_C_ 35.1), C-17 (*δ*_C_ 44.0), C-5 (*δ*_C_ 54.4), C-16 (*δ*_C_ 74.7), C-2 (*δ*_C_ 145.6) located the quaternary carbon of C-16 (Fig. 2), indicating that **3** was 16-hydroxy ibogamine. According to the biogenetic pathway of iboga-type monoterpenoid indole alkaloids and molecular model, the substituent of C-16 was *α*-oriented. Therefore, the structure of **3**, 16*α*-hydroxyl-ibogamine (**3**), was defined as shown in Fig. 2.

Compounds **1**–**9** were evaluated for their cytotoxicity activity based on the MTT method (camptothecin was used as a positive control). However, none of these compounds showed any significant activity against SGC-7901 gastric cancer, HeLa, and A-549 lung cancer cell lines (IC_50_ > 20 µM).

## Experimental

### General Experimental Procedures

Optical rotations were measured with a JASCO P-1020 digital polari-meter (Horiba, Kyoto, Japan). UV spectra were recorded on a Shimadzu UV2401PC spectrometer (Shimadzu, Kyoto, Japan). NMR spectra were performed on a Bruker AVANCE III 400 MHz spectrometers (Bruker Biospin GmbH, Karlsruhe, Germany) and a Bruker AVANCE III 500 MHz spectrometers (Bruker Biospin GmbH, Karlsruhe, Germany). HRESIMS data were acquired on a Shimadzu UPLC-IT-TOF spectrometer (Shimadzu, Kyoto, Japan). HPLC analyses were performed on a Waters instrument consisting of a Waters 1525EF pump coupled with a Waters 2998 photodiode array detector and a Waters fraction collector III. The analytical separations were performed on a Sunfire C_18_ column (5 μm, 150 mm × 4.6 mm). Preparative separations were done a Sunfire C_18_ column (5 μm, 250 mm × 19 mm). The following stationary phases were used for column chromatography (CC): silica gel (200–300 mesh, Qingdao Marine Chemical Ltd., Qingdao, China), C_18_ silica gel (50 μm, YMC Co. Ltd., Japan), and Sephadex LH-20 (Mitsubishi Co., Ltd., Japan). Each separation step was monitored by TLC on silica gel plates (GF254, Qingdao Marine Chemical Co., Ltd., Qingdao, China), and spots were visualized using Dragendorff’s reagent. MPLC was performed using a Buchi pump system coupled with RP-18 silica gel-packed glass columns (15 × 230 and 26 × 460 mm, respectively).

### Plant Material

Leaves and branches of *Tabernaemontana divaricata* were collected in Hainan Province, P. R. China, and identified by Dr. Sheng-Zhou Huang. A voucher specimen (Cai20150424) was deposited in the State Key Laboratory of Phytochemistry and Plant Resources in West China, Kunming Institute of Botany, Chinese Academy of Sciences.

### Extraction and Isolation

Air-dried branches and leaves of *T. divaricata* (46 kg) were powdered and extracted with MeOH (96 h × 3) at room temperature. The extract (2.5 kg) was partitioned between 0.5% HCl solution and EtOAc, and the acidic water layer was adjusted to pH 8–9 with 15% ammonia solution and subsequently extracted with EtOAc. This yielded 325 g crude alkaloid extract. This extract was subjected to column chromatography (CC) over silica gel and eluted with gradient CHCl_3_–Me_2_CO (1:0–1:1, v/v) to afford five fractions (I–V).

Fraction II (20.3 g) was further chromatographed on a C_18_ MPLC column eluted with a gradient of MeOH–H_2_O (30:70–100:0, v/v). Six subfractions II-1 to II-6 were collected from this procedure. Subfraction II-2 was subjected to C_18_ MPLC column once again using MeOH–H_2_O (20:80–70:30, v/v) as eluent which yielded seven subfractions (II-2-1 to II-2-7). Subfraction II-2-5 was refined by a preparative C_18_ HPLC column using a gradient of MeCN–H_2_O (45:55–60:40, v/v). This afforded 4.0 mg of **1**. Fraction II-2-7 was further purified by preparative HPLC with a gradient from 50 to 65% aqueous acetonitrile. This afforded 6.7 mg of **8**. Fraction II-3 was separated by reversed phase MPLC column with a gradient of MeOH–H_2_O (40:60–85:15, v/v). This step afforded five subfractions (II-3-1 to II-3-5). Fraction II-3-5 was separated by prep. HPLC with a gradient of MeOH–H_2_O (65:35–80:20, v/v). This step yielded 8.2 mg of compound **9**. Fraction II-4 was separated using a Sephadex LH-20 column eluted with MeOH. Four subfractions (II-4-1 to II-4-4) were collected. Fraction II-4-2 was separated on a prep. C_18_ HPLC using a gradient of MeCN–H_2_O (55:45–70:30, v/v) which afforded 6.7 mg of **6**. Fraction II-4-3 was subjected to a prep. C_18_ HPLC eluted with a gradient of MeOH–H_2_O (75:25–90:10, v/v). This step generated 2.2 mg of **4**. Compound **7** (1.5 g) was crystalized from fraction II-5. Subfraction II-6 was subjected to C_18_ MPLC eluted with mixtures of MeOH and water (60:40–90:10, v/v). Five subfractions (II-6-1 to II-6-5) were collected. Fraction II-6-2 was further separated using reversed phase MPLC with a gradient of MeOH (from 60 to 85%) in H_2_O, affording one subfraction (II-6-2-4). Refining of this subfraction by prep. HPLC with a gradient of MeCN–H_2_O (65:35–80:20, v/v) gave 30.9 mg of **5**. Fraction II-6-5 was chromatographed over Sephadex LH-20 column eluted with MeOH. This generated five subfractions (II-6-5-1 to II-6-5-5). Fraction II-6-5-1 was separated by prep. HPLC using a gradient of H_2_O (from 70 to 85%) in MeCN. This step yielded 7.1 mg of **2** and 5.7 mg of **3**.

#### 3*α*-Hydroxymethyl-ibogamine (**1**)

Pale yellow amorphous powder, [*α*]_D_^17.5^ −29.9 (*c* 0.13, MeOH); UV (MeOH) λ_max_ (log ε) 196 (4.10), 214 (4.30), 227 (4.41), 284 (3.75) nm; ^1^H and ^13^C NMR data, see Table 1. HRESIMS *m/z* 311.2118 [M + H]^+^ (calcd. for C_20_H_26_N_2_O, 311.2118).

#### 3*α*-acetatemethoxyl-ibogamine (**2**)

Pale yellow amorphous powder, [*α*]_D_^17.5^ −15.0 (*c* 0.11, MeOH); UV (MeOH) λ_max_ (log ε) 211 (4.26), 227 (4.40), 285 (3.74) nm; ^1^H and ^13^C NMR data, see Table 1. HRESIMS *m/z* 353.2223 [M + H]^+^ (calcd. for C_22_H_28_N_2_O_2_, 353.2224).

#### 16*α*-hydroxyl-ibogamine (**3**)

Pale yellow amorphous powder, [*α*]_D_^17.5^ −19.2 (*c* 0.07, MeOH); UV (MeOH) λ_max_ (log ε) 211 (4.09), 223 (4.10), 285 (3.49) nm; ^1^H and ^13^C NMR data, see Table 1. HRESIMS *m/z* 297.1964 [M + H]^+^ (calcd. for C_19_H_24_N_2_O, 297.1961).

### Cytotoxicity

The human A-549 lung cancer, SGC-7901 gastric cancer, and HeLa cell lines were used in the performed cytotoxic assay. These cells were grown in DMEM media (HyClone, USA) supplemented with 10% fetal bovine serum (HyClone, USA) at 37 °C in 5% CO_2_. The cytotoxicity of all alkaloids was determined based on the MTT method in 96-well microplates. In short, 100 µL adherent cells were seeded into each well and incubated for 12 h before the addition of the test alkaloids/drug. At the same time, the suspended cells were seeded at an initial density of 1 × 10^5^ cells/mL just before addition of the purified compounds. Each tumor cell line was exposed to a single test compound at concentrations of 0.8, 4 and 20 μM in DMSO. Camptothecin was used as positive control. Each test was performed in triplicate. After treatment, cell viability was assessed, cell growth graphed and IC_50_ values were calculated using Reed and Muench’s method [15].

## Electronic supplementary material

Below is the link to the electronic supplementary material.

Supplementary material 1 (PDF 1986 kb) 1D and 2D NMR spectra, and HRESIMS of compounds **1**–**3** are available as Supplementary Information
