# Supplementary material for: Three New Indole Alkaloids from Tabernaemontana divaricata
Source: Nat Prod Bioprospect. 2018 May 12;8(3):183–8. doi: 10.1007/s13659-018-0166-x (PMC5971036; doi:10.1007/s13659-018-0166-x)
Supplement: Supplementary file 1 — Supplementary material 1 (PDF 1986 kb) 1D and 2D NMR spectra, and HRESIMS of compounds 1–3 are available as Supplementary Information [file 13659_2018_166_MOESM1_ESM.pdf]

SUPPORTING INFORMATION

Three new indole alkaloids from *Tabernaemontana divaricata*

Yan Deng,[a,b,c] Mei-Fen Bao,[a,c] Bao-Bao Shi,[a,b,c], Jing Wu,[a,c] Xiang-Hai Cai*,[a,c]

[a]State Key Laboratory of Phytochemistry and Plant Resources in West China, Kunming Institute of Botany, Chinese Academy of Sciences, Kunming 650201, People's Republic of China.

[b]University of Chinese Academy of Sciences, Beijing 100049, People's Republic of China

[c]Yunnan Key Laboratory of Natural Medicinal Chemistry, Kunming 650201, People's Republic of China.

*Corresponding Author. Tel: +86-871-65223242; Fax: +86-871-65150227.

E-mail: xhcai@mail.kib.ac.cn.

Content:

Figure S1-S6. NMR spectra of 3α-hydroxymethyl-ibogamine (**1**)--------------3

Figure S7-S12. NMR spectra of 3α-acetatemethoxyl-ibogamine (**2**) ----------9

Figure S13-S17. NMR spectra of 16α-hydroxyl-ibogamine (**3**)-----------------15

Figure **S1**. ^{1}H NMR spectrum of 3α-hydroxymethyl-ibogamine (**1**)

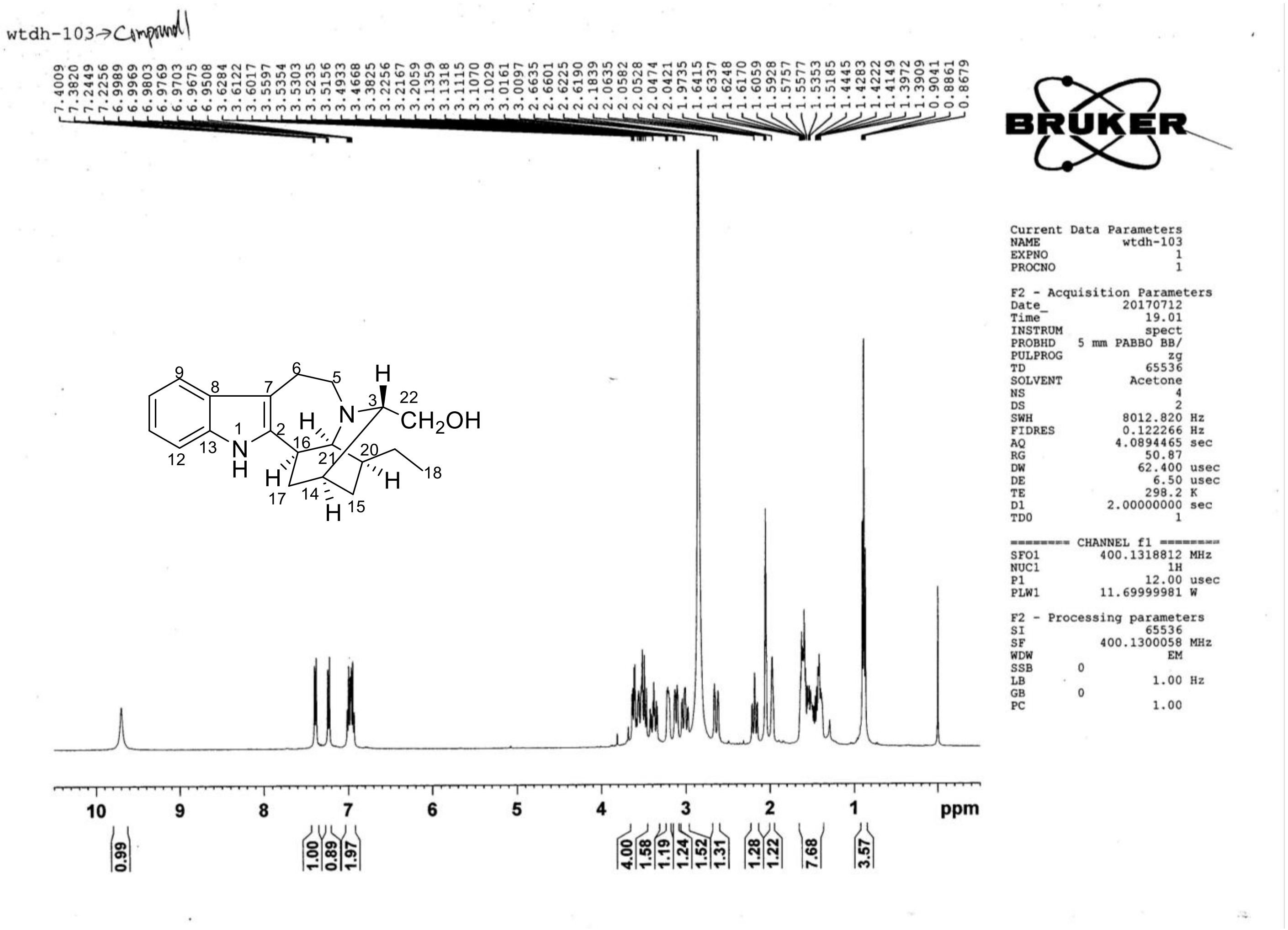

Figure **S2**. ^{13}C NMR and DEPT spectra of 3α-hydroxymethyl-ibogamine (**1**)

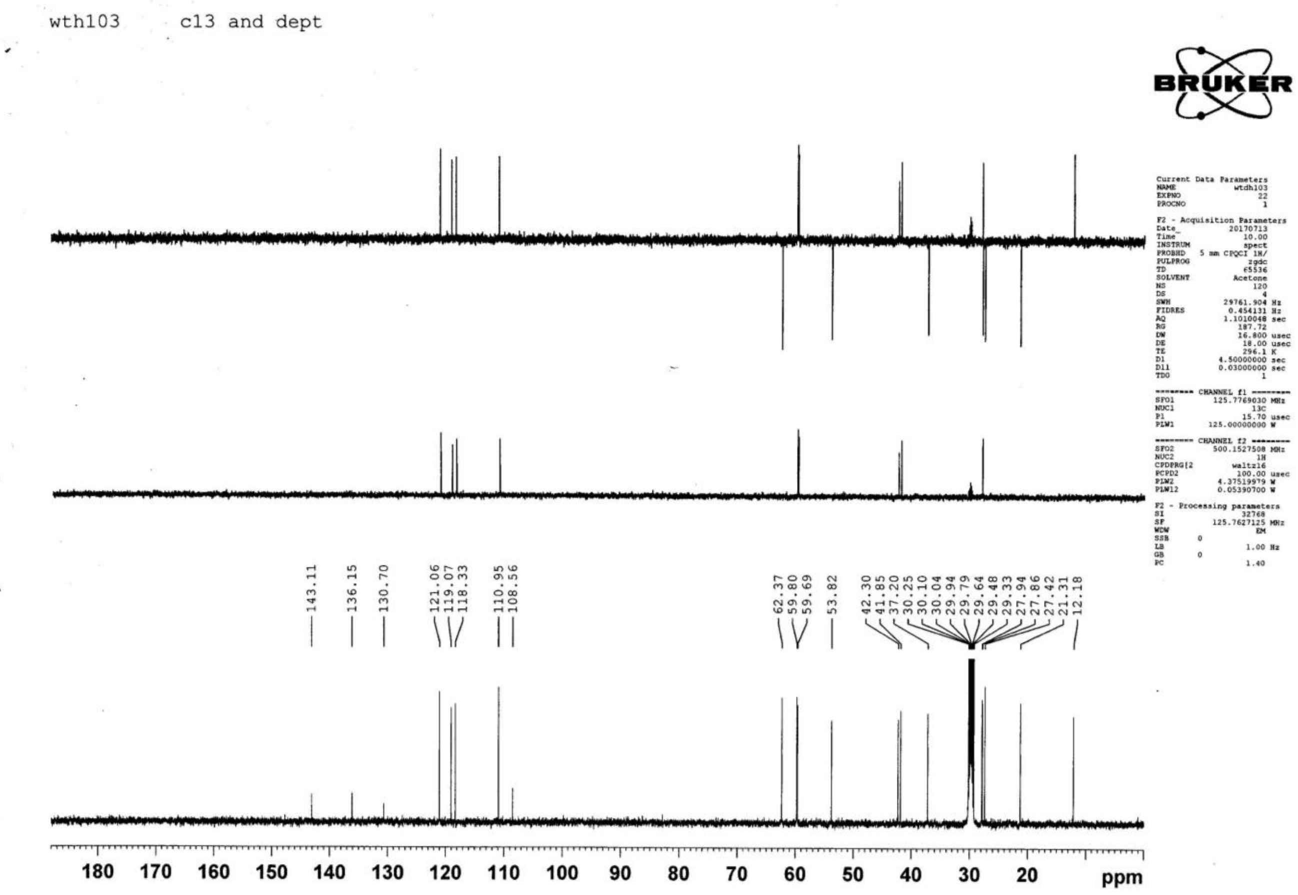

Figure **S3**. HSQC spectrum of 3α-hydroxymethyl-ibogamine (**1**)

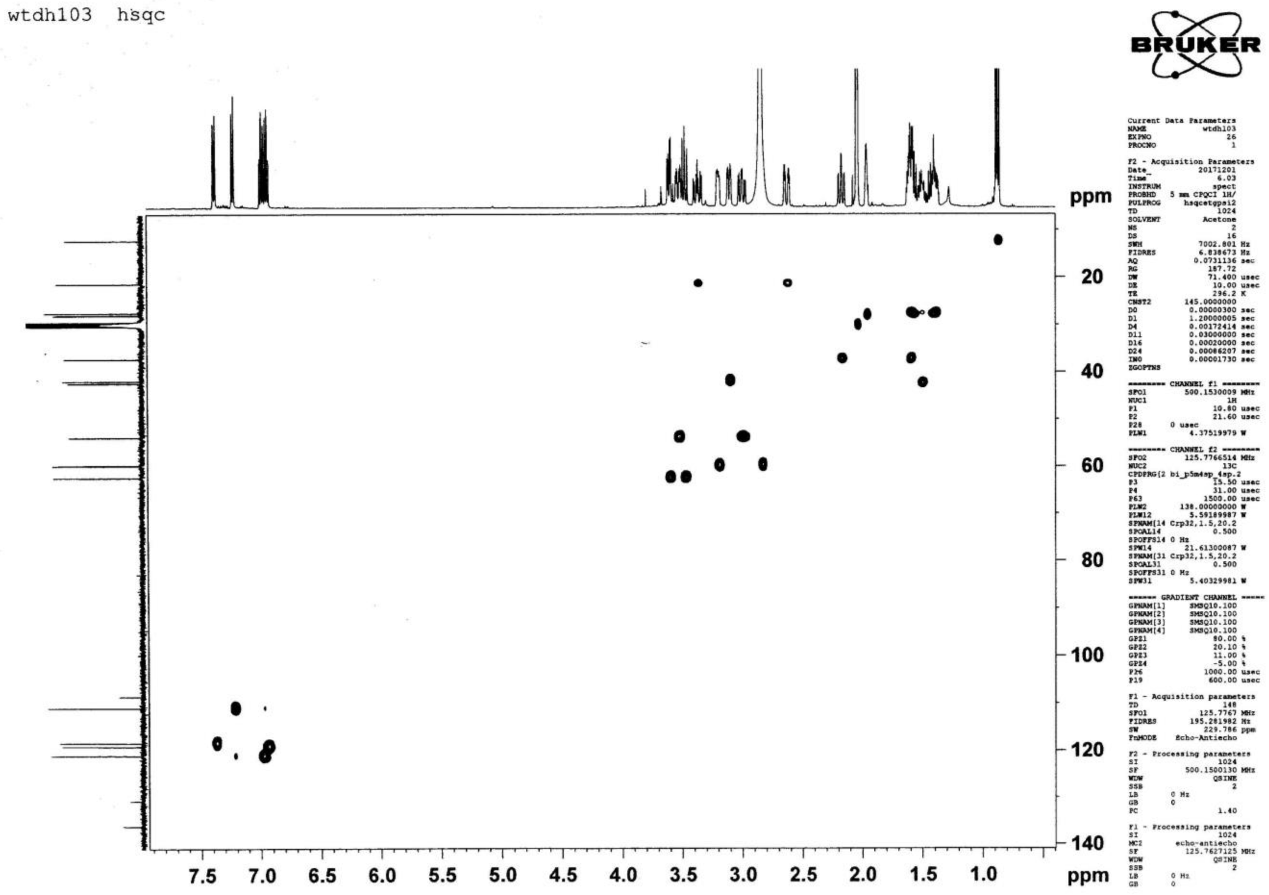

Figure **S4**. HMBC spectrum of 3α-hydroxymethyl-ibogamine (**1**)

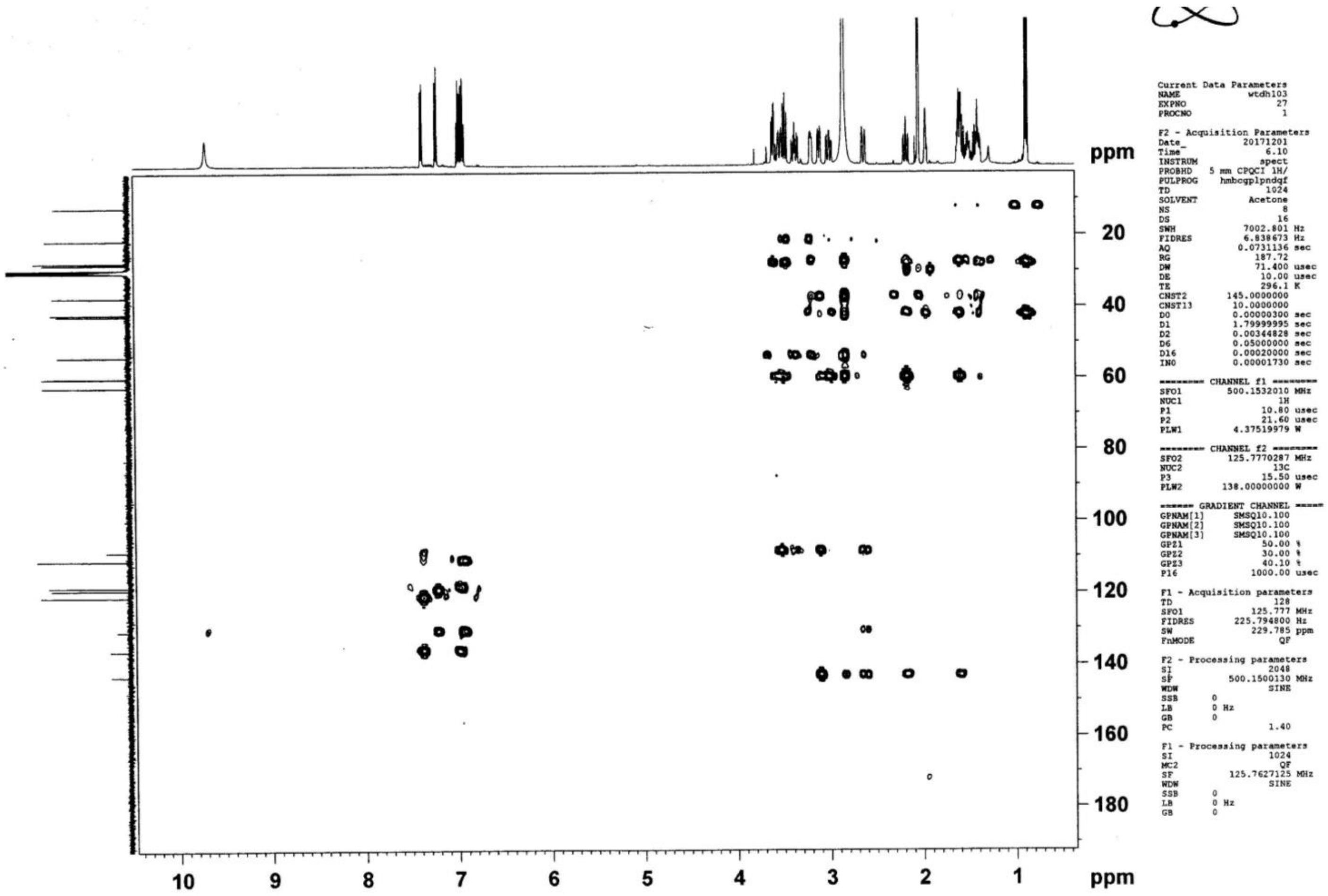

Figure **S5**. ROESY spectrum of 3α-hydroxymethyl-ibogamine (**1**)

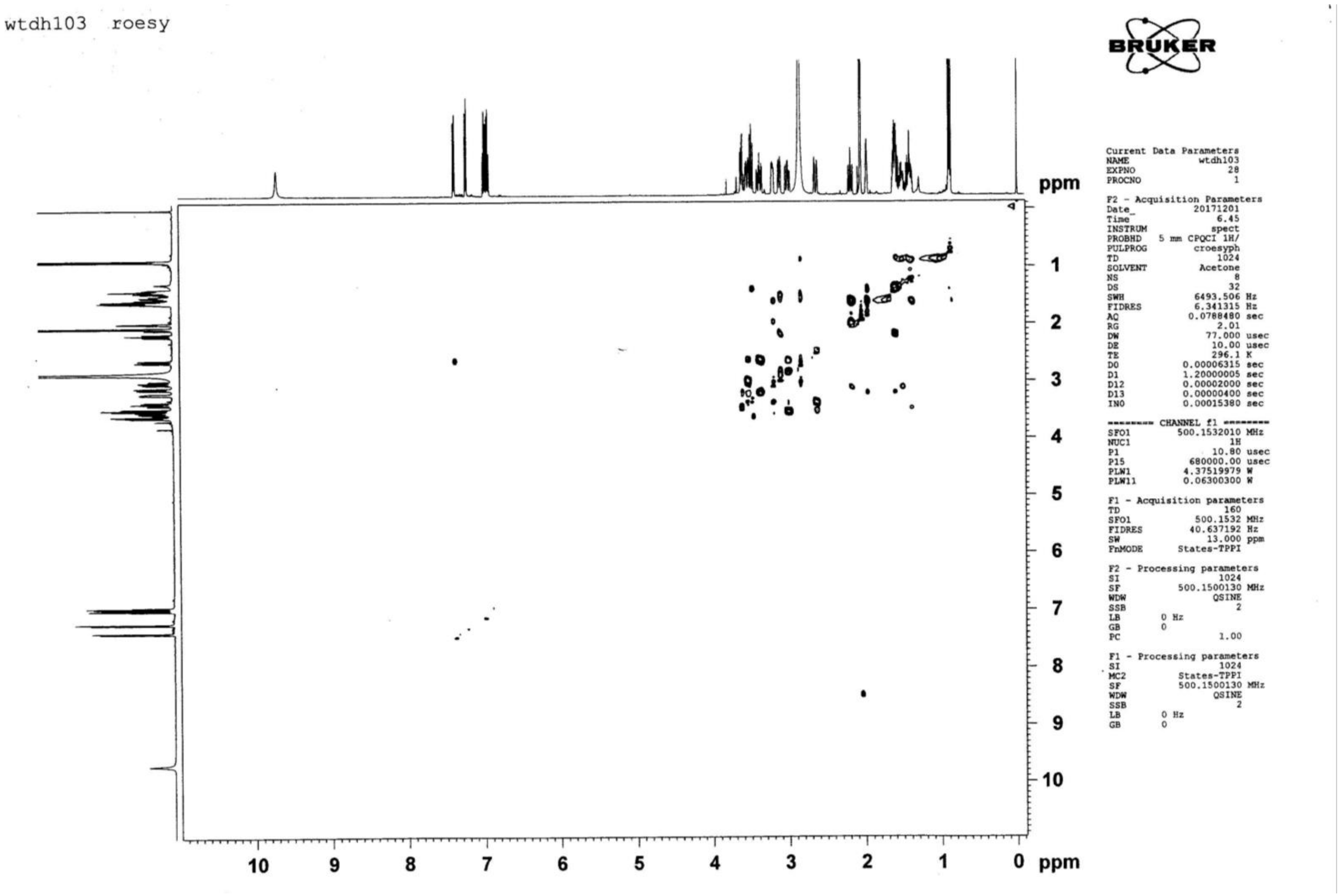

Figure **S6**. HRESIMS spectrum of 3α-hydroxymethyl-ibogamine (**1**)

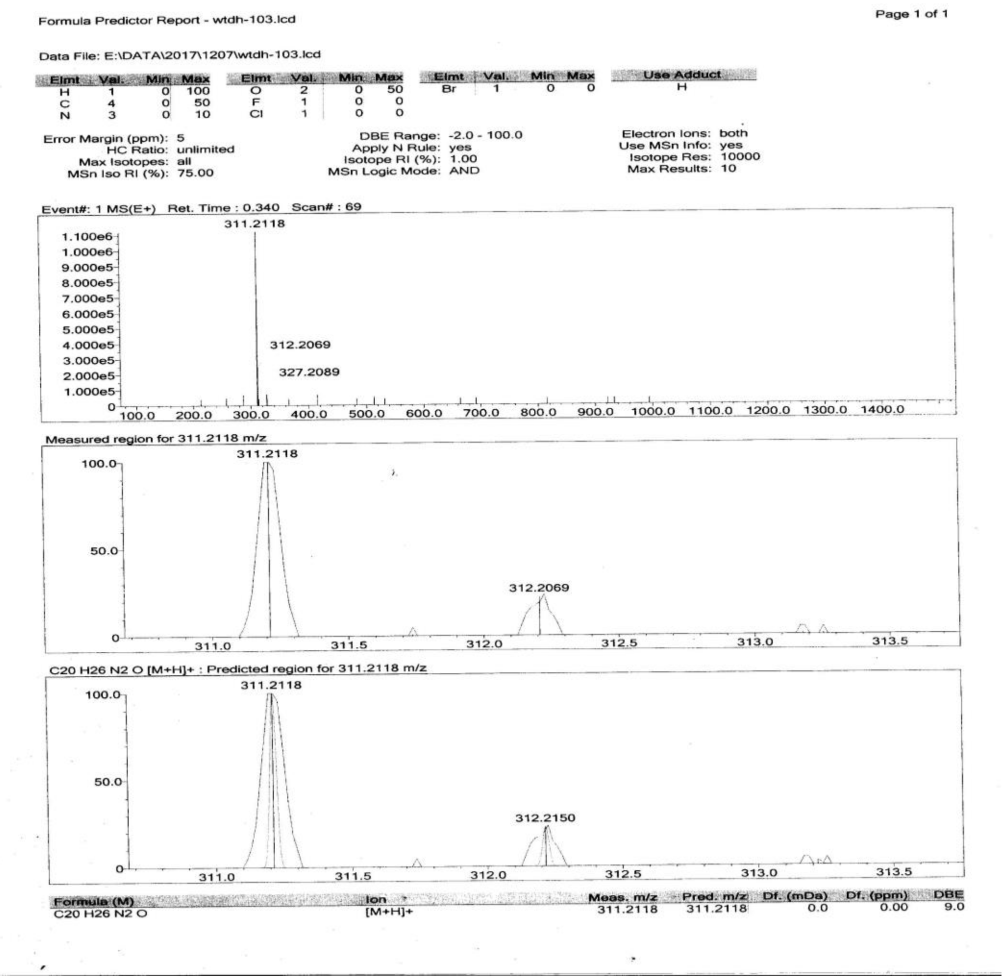

Figure **S7**. ^{1}H NMR spectrum of 3α-acetatemethoxyl-ibogamine (**2**)

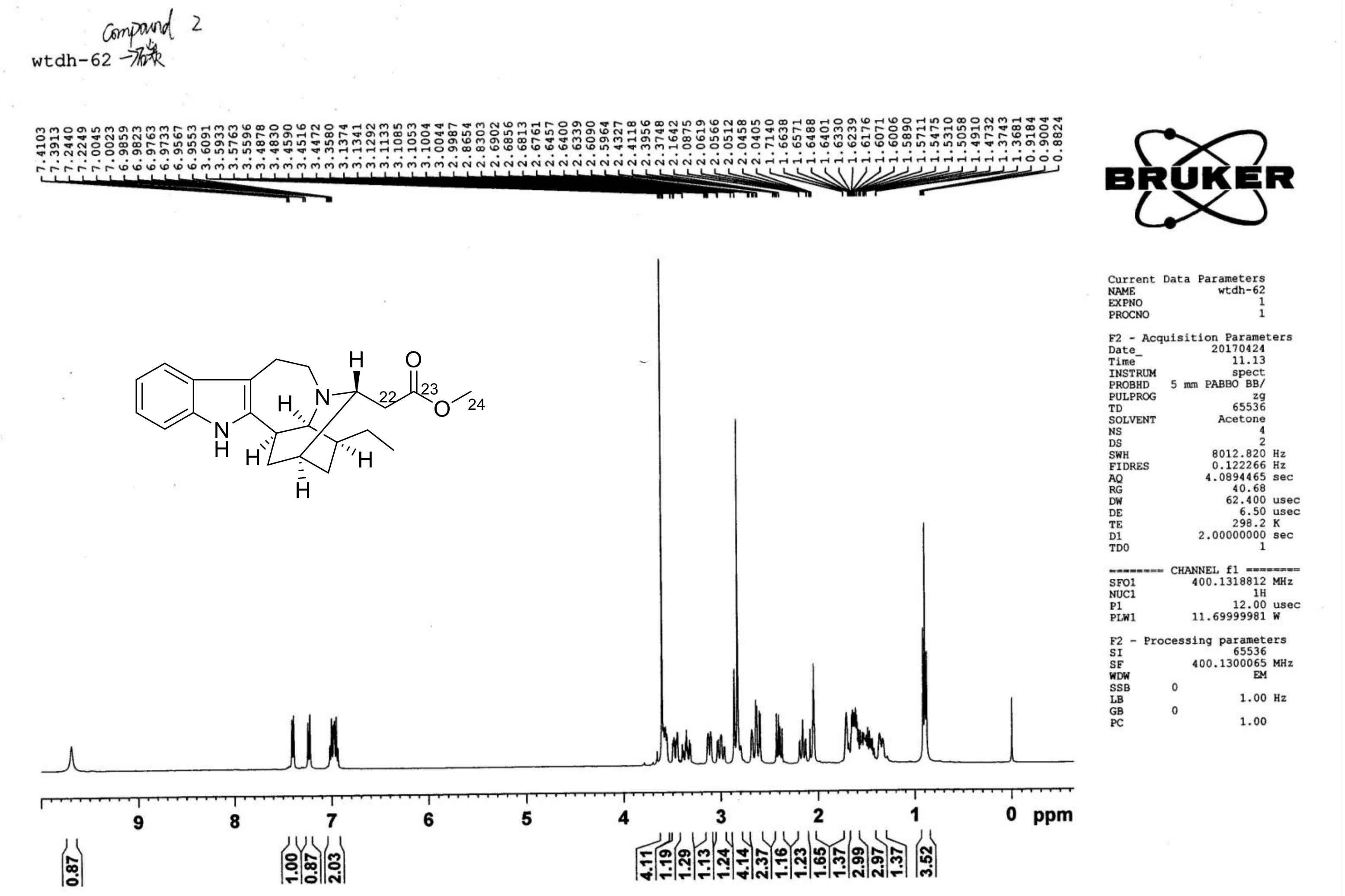

Figure **S8**. ^{13}C NMR and DEPT spectra of 3α-acetatemethoxyl-ibogamine (**2**)

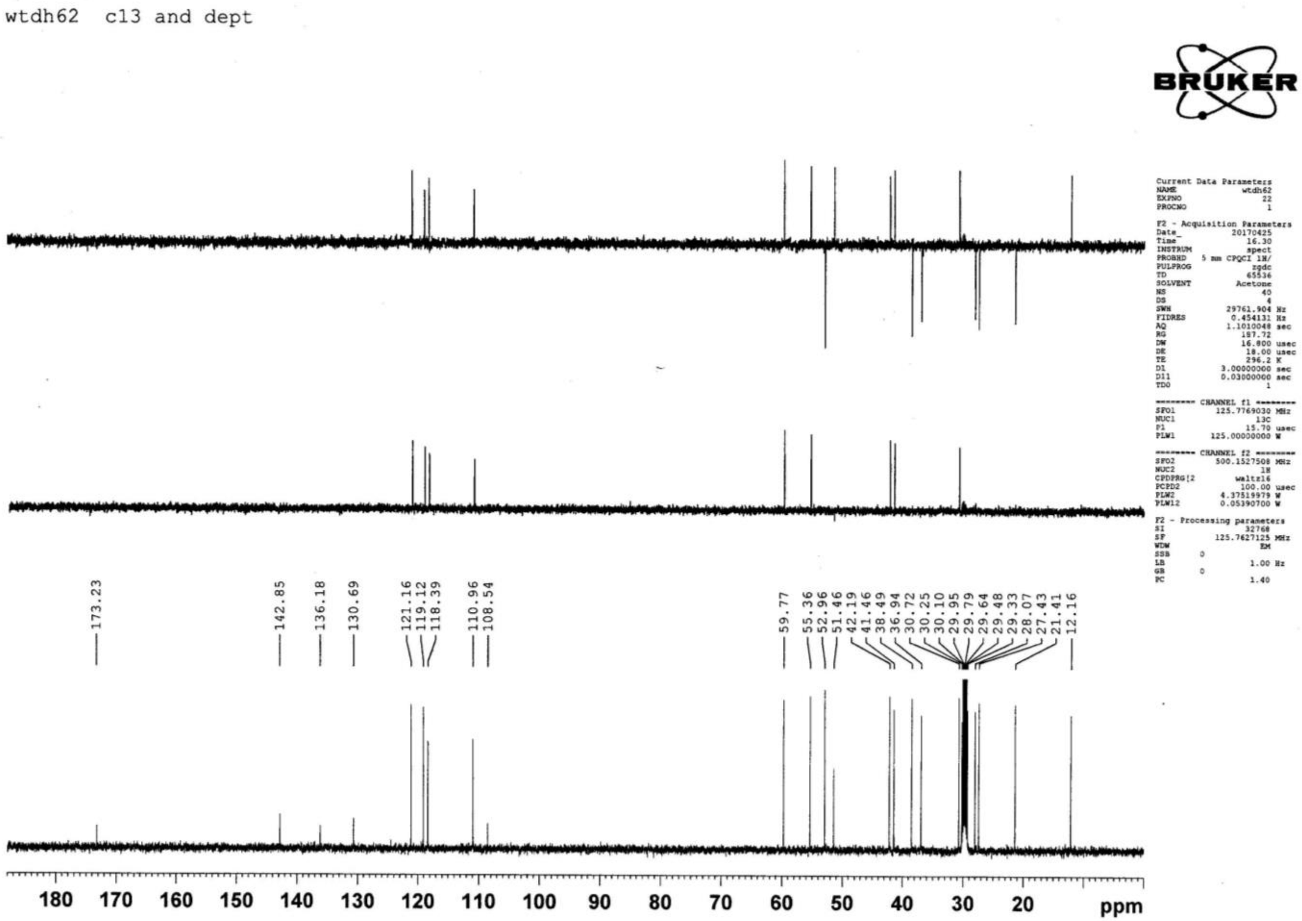

Figure **S9**. HSQC spectrum of 3α-acetatemethoxyl-ibogamine (**2**)

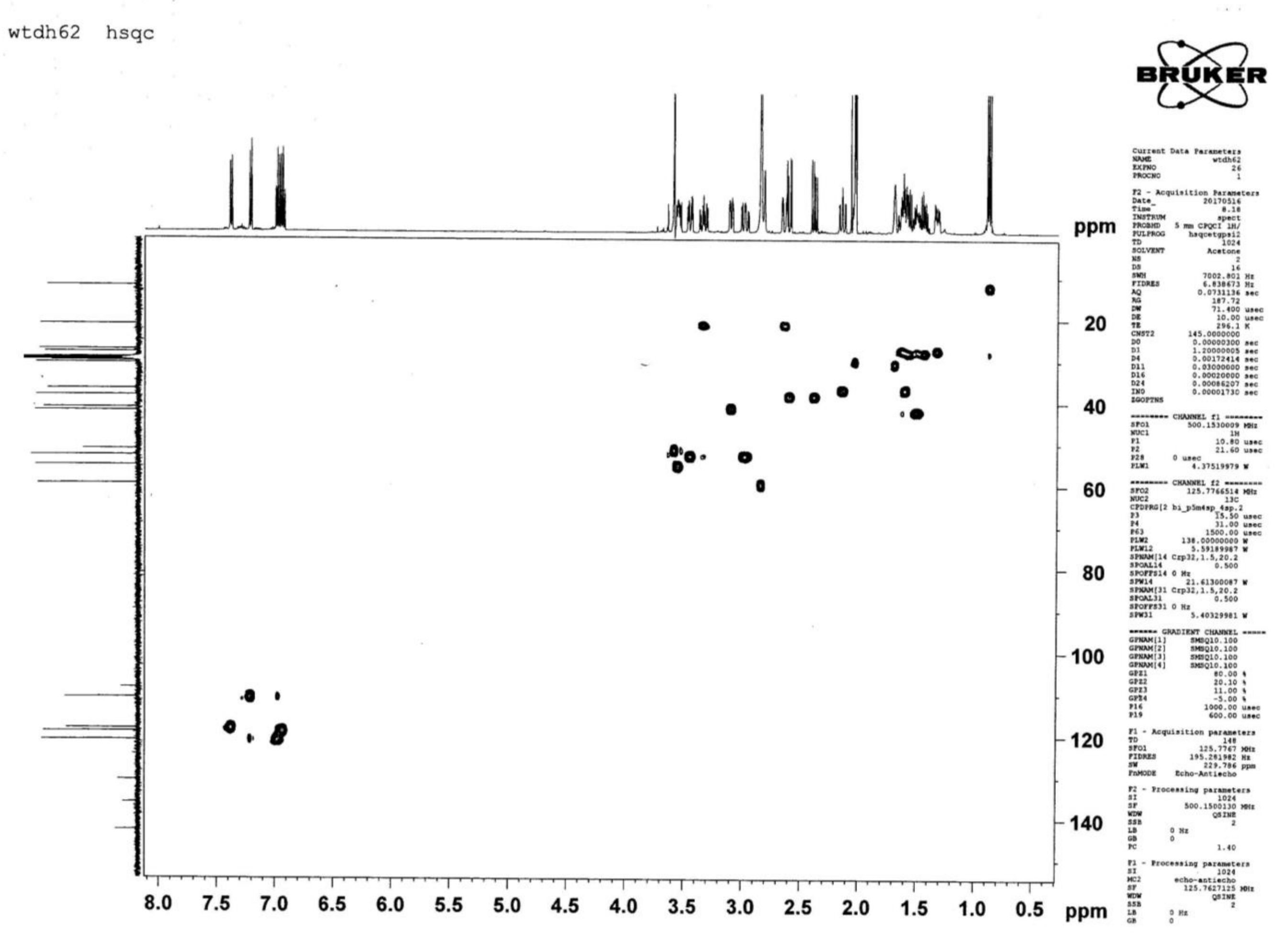

Figure **S10**. HMBC spectrum of 3α-acetatemethoxyl-ibogamine (**2**)

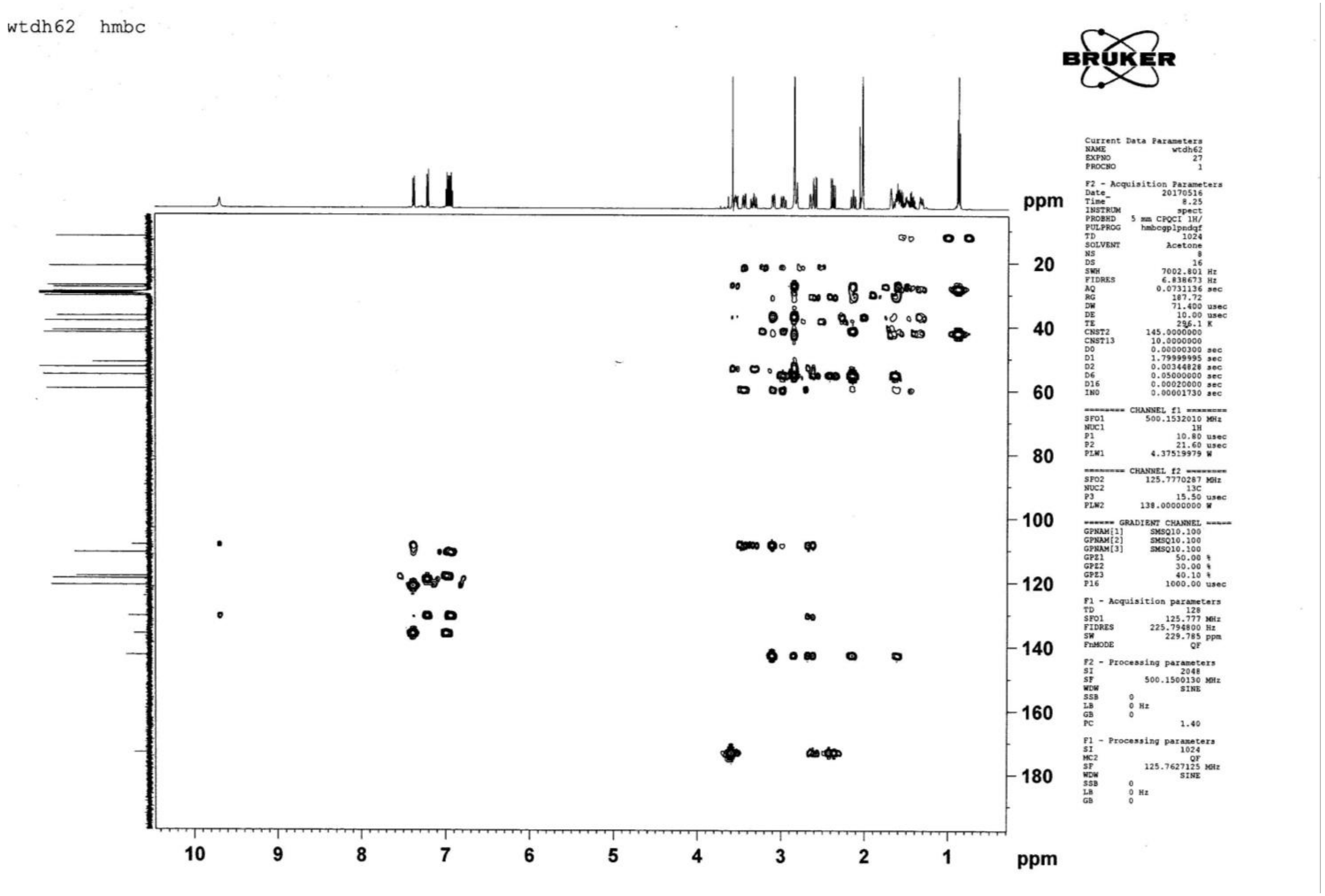

Figure **S11**. ROESY spectrum of 3α-acetatemethoxyl-ibogamine (**2**)

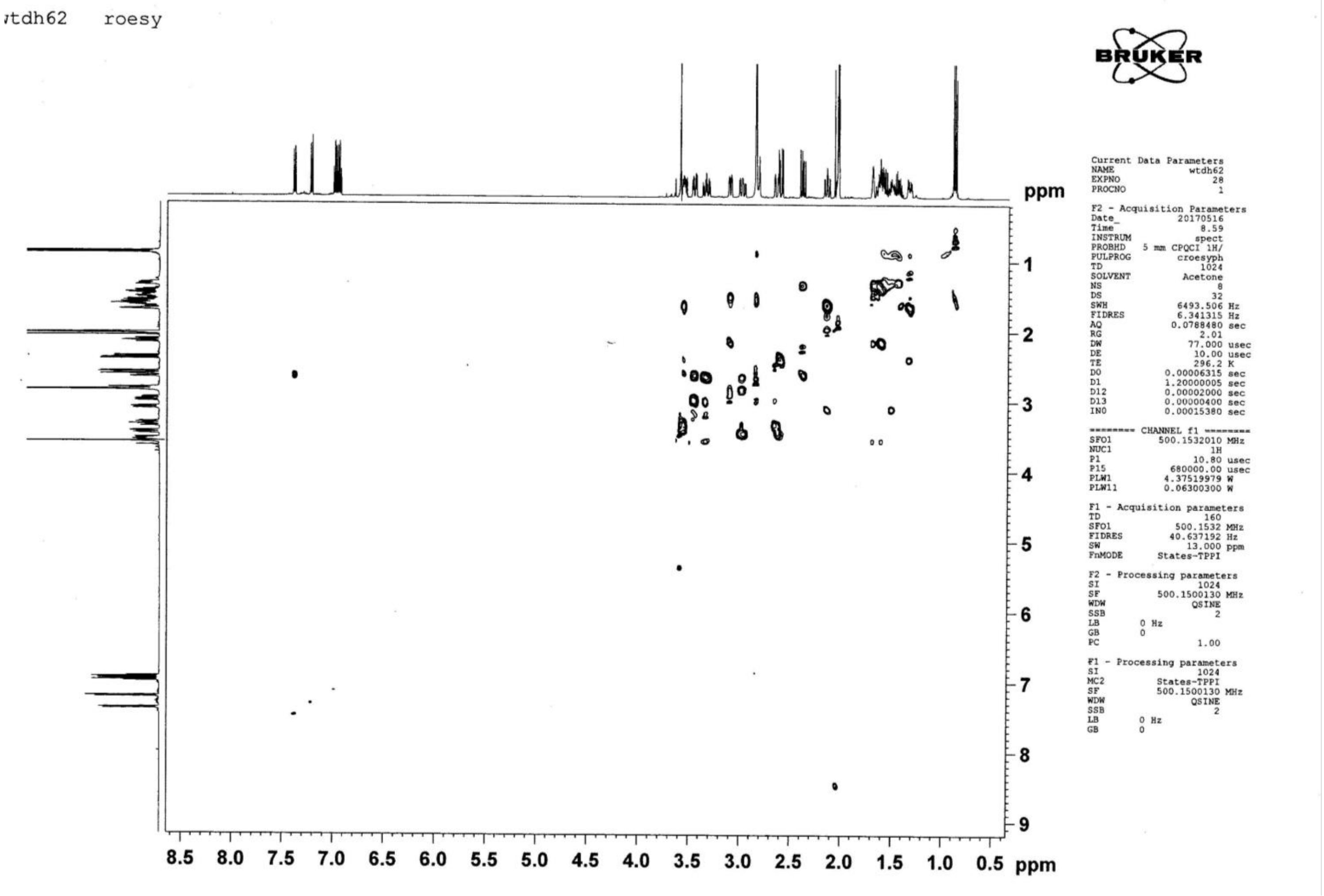

Figure **S12**. HRESIMS spectrum of 3α-acetatemethoxyl-ibogamine (**2**)

Formula Predictor Report - wtdh-62.lcd

Page 1 of 1

Data File: E:\DATA\2017\1207\wtdh-62.lcd

Elmt	Val.	Min	Max	Elmt	Val.	Min	Max	Elmt	Val.	Min	Max	Use Adduct
H	1	0	100	O	2	0	50	Br	1	0	0	H
C	4	0	50	F	1	0	0					
N	3	0	10	Cl	1	0	0					

Error Margin (ppm): 5
HC Ratio: unlimited
Max Isotopes: all
MSn Iso RI (%): 75.00

DBE Range: -2.0 - 100.0
Apply N Rule: yes
Isotope RI (%): 1.00
MSn Logic Mode: AND

Electron Ions: both
Use MSn Info: yes
Isotope Res: 10000
Max Results: 10

Event#: 1 MS(E+) Ret. Time : 0.360 Scan# : 73

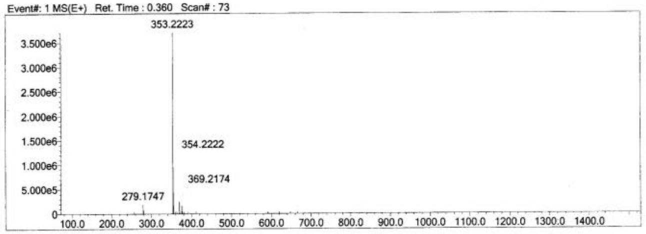

Measured region for 353.2223 m/z

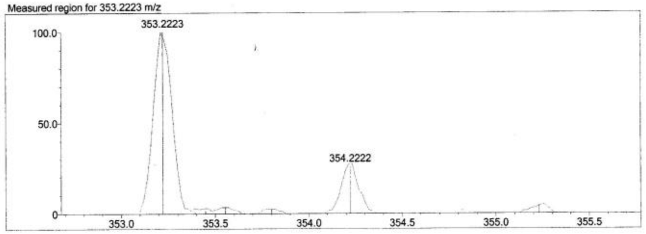

C22 H28 N2 O2 [M+H]+ : Predicted region for 353.2224 m/z

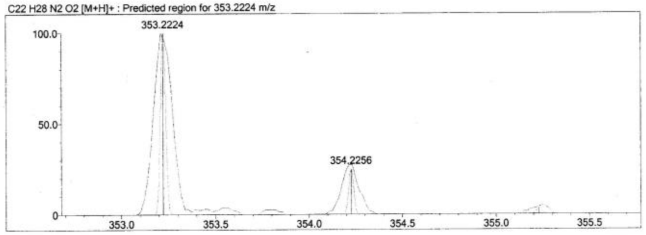

Formula (M)	Ion	Meas. m/z	Pred. m/z	Df. (mDa)	Df. (ppm)	DBE
C22 H28 N2 O2	[M+H]+	353.2223	353.2224	-0.1	-0.28	10.0

Figure **S13**. ^{1}H NMR spectrum of 16α-hydroxyl-ibogamine (**3**)

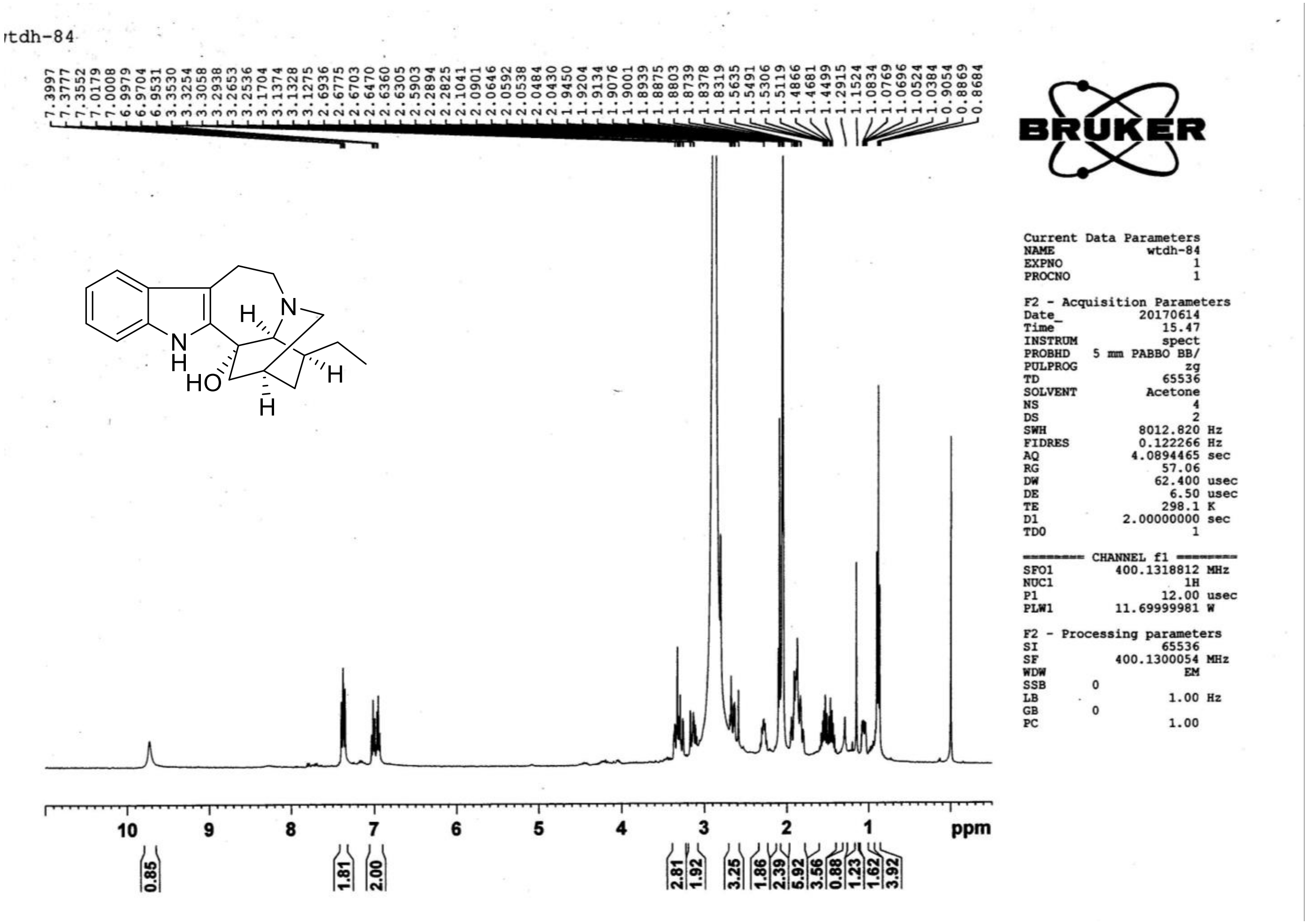

Figure **S14**. ^{13}C NMR and DEPT spectra of 16α-hydroxyl-ibogamine (**3**)

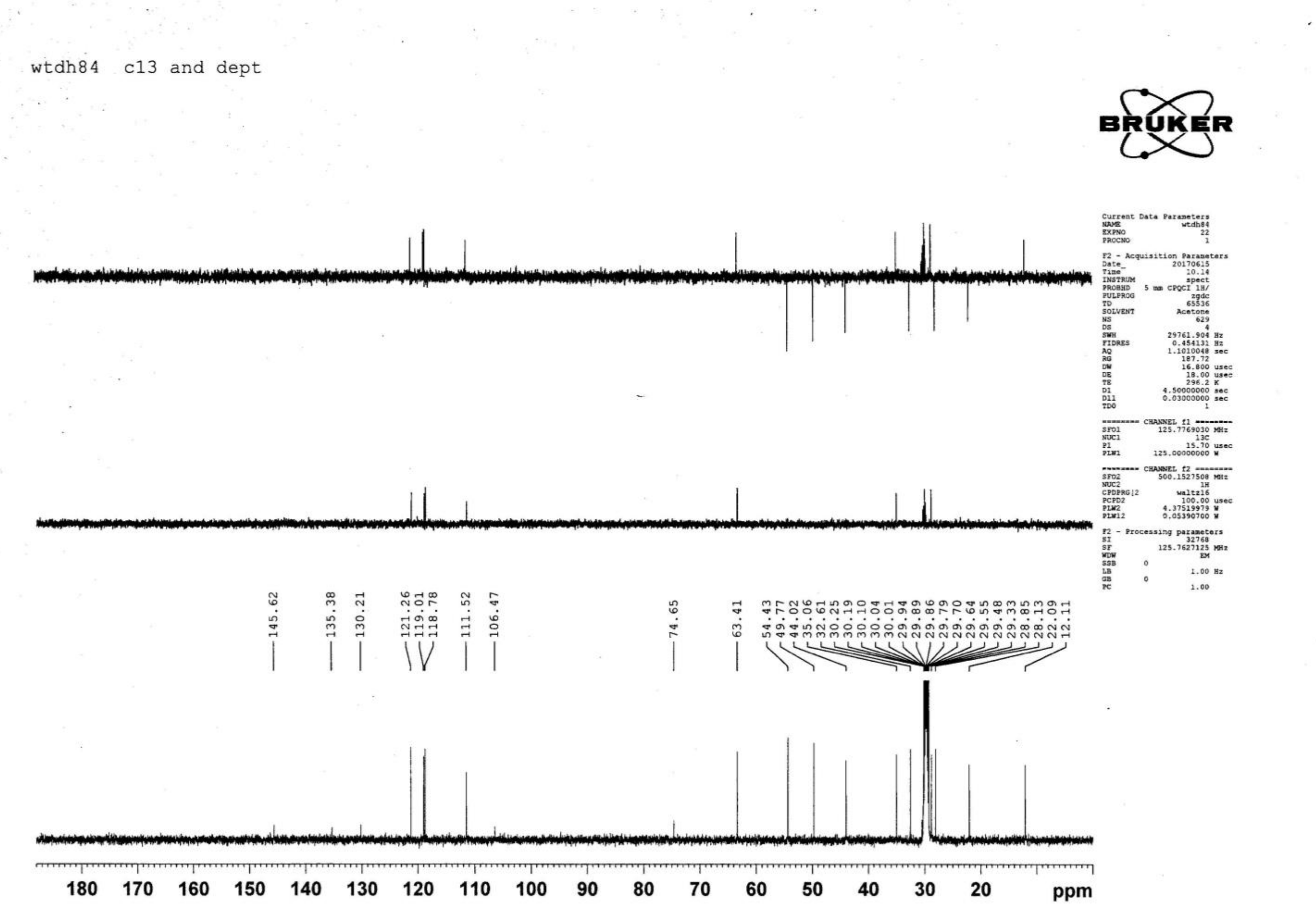

Figure **S15**. HSQC spectrum of 16α-hydroxyl-ibogamine (**3**)

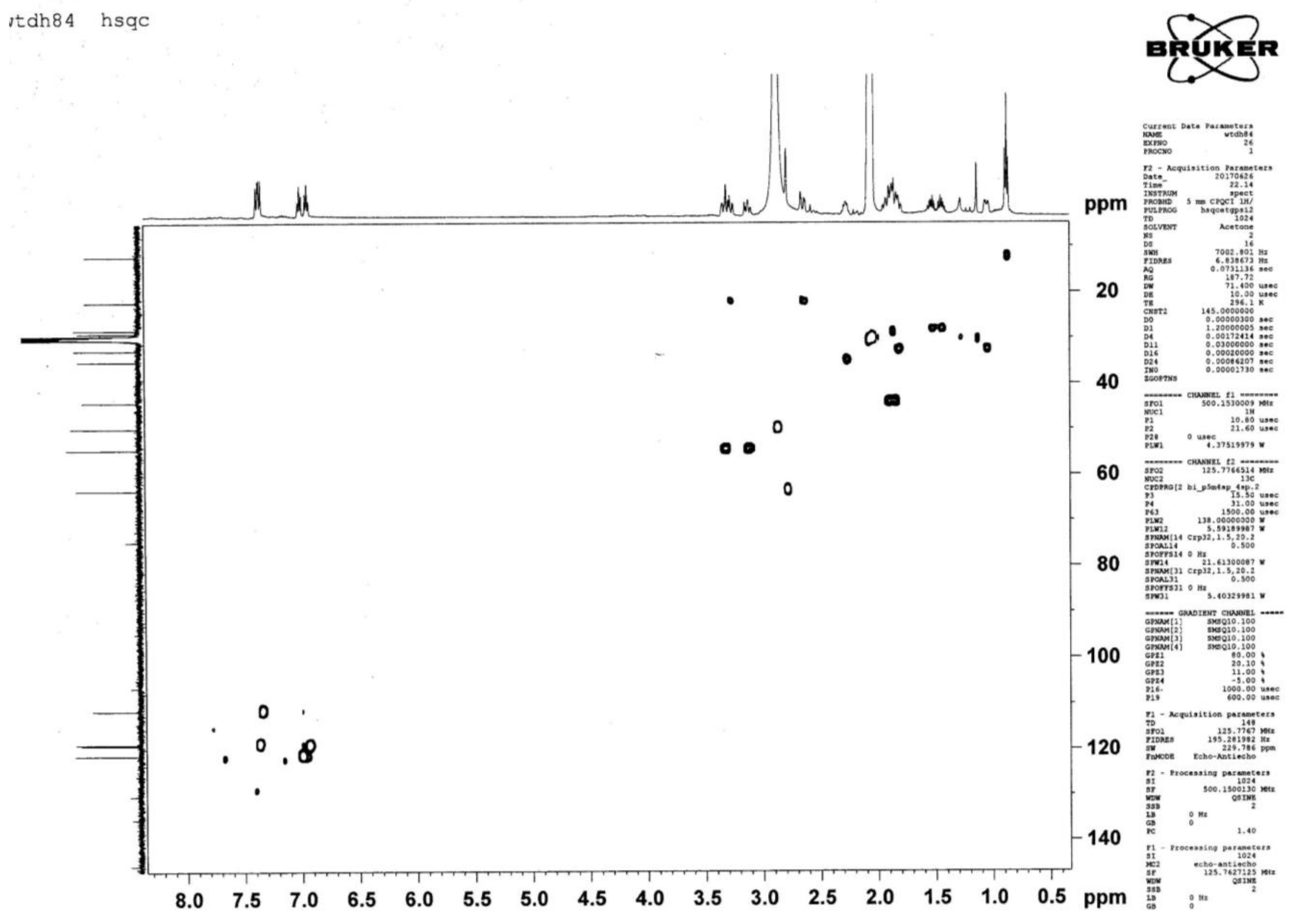

Figure **S16**. HMBC spectrum of 16α-hydroxyl-ibogamine (**3**)

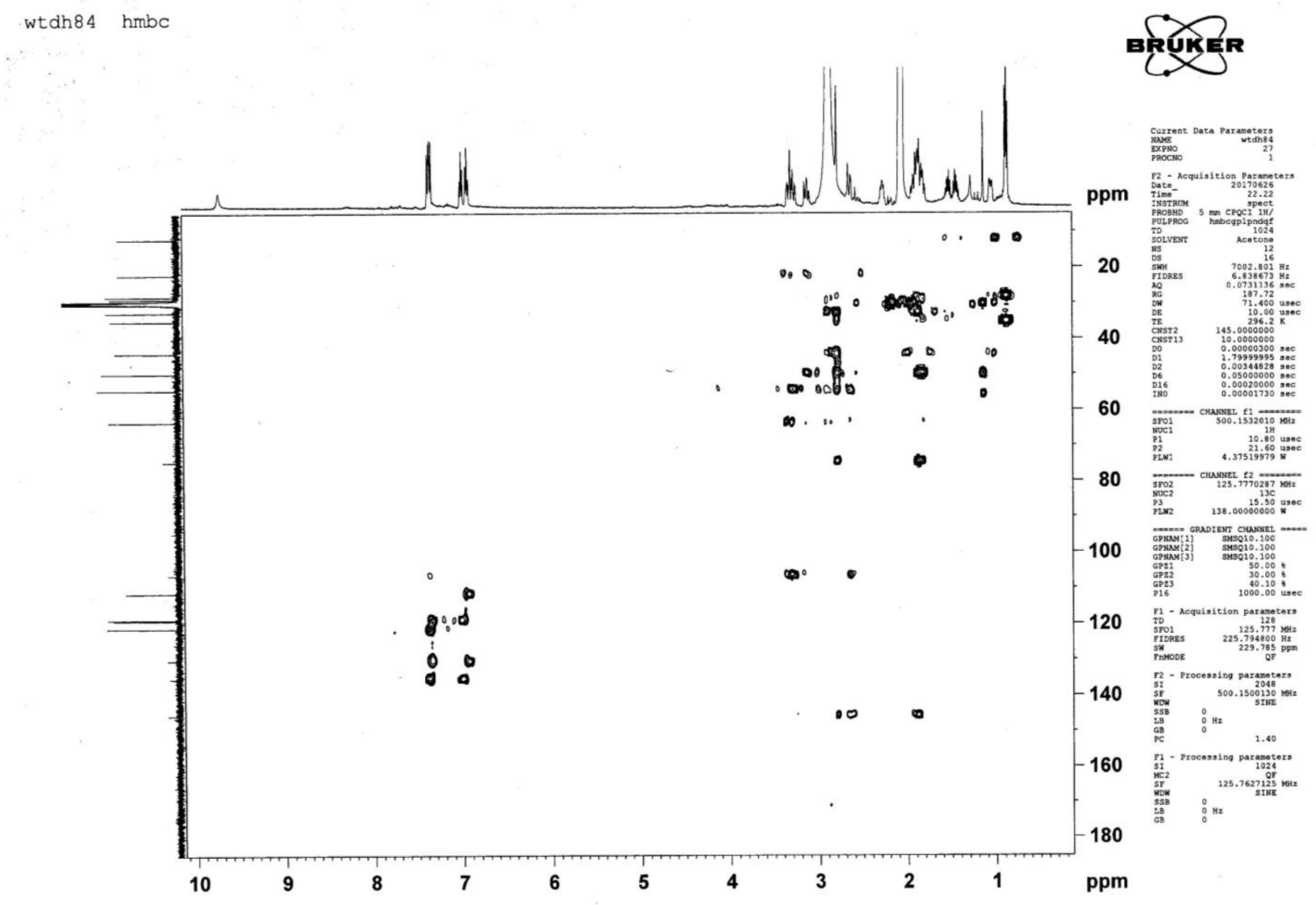

Figure **S17**. HRESIMS spectrum of 16α-hydroxyl-ibogamine (**3**)

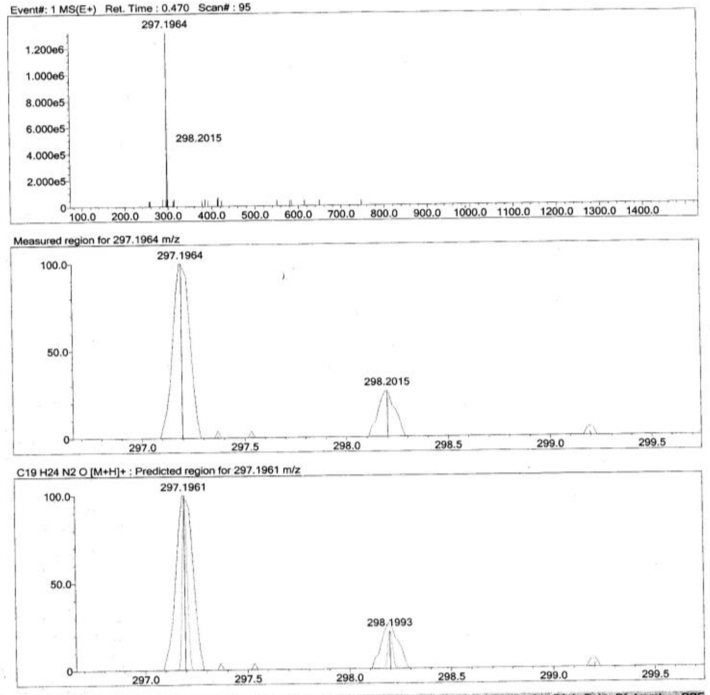
Formula Predictor Report - wtdh-84.lcd

Page 1 of 1

Data File: E:\DATA\2017\1207\wtdh-84.lcd

Elmt	Val.	Min	Max	Elmt	Val.	Min	Max	Elmt	Val.	Min	Max	Use Adduct
H	1	0	100	O	2	0	50	Br	1	0	0	H
C	4	0	50	F	1	0	0					
N	3	0	10	Cl	1	0	0					

Error Margin (ppm): 5
HC Ratio: unlimited
Max Isotopes: all
MSn Iso RI (%): 75.00

DBE Range: -2.0 - 100.0
Apply N Rule: yes
Isotope RI (%): 1.00
MSn Logic Mode: AND

Electron Ions: both
Use MSn Info: yes
Isotope Res: 10000
Max Results: 10

Formula (M)	Ion	Meas. m/z	Pred. m/z	Df. (mDa)	Df. (ppm)	DBE
C19 H24 N2 O	[M+H]+	297.1964	297.1961	0.3	1.01	9.0
